# Collecting Duct Renin Does Not Mediate DOCA-Salt Hypertension or Renal Injury

**DOI:** 10.1371/journal.pone.0159872

**Published:** 2016-07-28

**Authors:** Kai Song, Deborah Stuart, Nikita Abraham, Fei Wang, Shuping Wang, Tianxin Yang, Curt D. Sigmund, Donald E. Kohan, Nirupama Ramkumar

**Affiliations:** 1 Division of Nephrology and Hypertension, University of Utah Health Sciences Center, Salt Lake City, Utah, United States of America; 2 Veterans Affairs Salt Lake City Health Care System, Salt Lake City, Utah, United States of America; 3 Department of Nephrology, Second Affiliated Hospital of Soochow University, Soochow City, China; 4 Department of Pharmacology, Roy J. and Lucille A. Carver College of Medicine, University of Iowa, Iowa City, Iowa, United States of America; University Medical Center Utrecht, NETHERLANDS

## Abstract

Collecting duct (CD)-derived renin is involved in the hypertensive response to chronic angiotensin-II (Ang-II) administration. However, whether CD renin is involved in Ang-II independent hypertension is currently unknown. To begin to examine this, 12 week old male and female CD-specific renin knock out (KO) mice and their littermate controls were subjected to uni-nephrectomy followed by 2 weeks of deoxycorticosterone acetate (DOCA) infusion combined with a high salt diet. Radiotelemetric blood pressure (BP) was similar between KO and control mice at baseline; BP increased in both groups to a similar degree throughout the 2 weeks of DOCA-salt treatment. Urinary albumin excretion and plasma blood urea nitrogen were comparable between the two groups after DOCA-salt treatment. Fibrosis as assessed by Masson’s Trichrome stain/Sirius Red stain and collagen-1 mRNA expression was similar between control and KO mice. Compared to baseline, DOCA-salt treatment decreased plasma renin concentration (PRC), urinary renin excretion and medullary renin mRNA expression in both floxed and CD renin KO mice with no detectable differences between the two groups. Further, in primary culture of rat inner medullary CD, aldosterone treatment did not change renin activity or total renin content. Taken together, these data suggest that CD derived renin does not play a role in DOCA-salt hypertension.

## Introduction

The renin angiotensin system (RAS) is an important modulator of blood pressure (BP) and sodium homeostasis. In recent years, there has been growing interest in the role of tissue-level RAS in BP regulation [1], among which the intra-renal RAS is of particular interest since it contains all elements necessary to generate tubular angiotensin-II (Ang-II) [2]. Apart from the juxta-glomerular apparatus, renin is synthesized in the connecting segment and collecting duct (CD) [2–4] and secreted into the lumen. Further, angiotensinogen and angiotensin converting enzyme are also expressed in the renal tubule leading to luminal Ang-II synthesis [5–8], which in turn can modulate electrolyte and water reabsorption and ultimately BP.

Previous studies suggest that Ang-II is a potent stimulant of CD renin synthesis [3, 4, 9, 10]. Chronic Ang-II infusion increases medullary renin levels and renin immunostaining in the CD [3, 9]. Similarly, medullary renin mRNA and protein levels are increased in 2-kidney, 1-clip Goldblatt hypertensive rats [11] and transgenic rats with inducible extra-renal mouse renin gene (*Ren2*) expression [12]. Further, CD renin fluorescence by multi-photon confocal microscopy and medullary renin activity is increased in streptozotocin-induced diabetic rats [13]. Collectively, these studies suggest that CD renin may be important in pathological states such as hypertension and diabetes. Notably, in contrast to JGA renin, renin synthesis in the CD is up regulated by systemic Ang-II [4].

To determine the role of CD renin in BP regulation, we developed gene-targeted mouse models with renin overexpression or ablation in the CD [14, 15]. Mice with CD specific overexpression of renin demonstrate modest salt sensitive hypertension and increased expression of membrane-bound epithelial Na channel (ENaC) [14]. In contrast, compared to controls, CD specific renin knock out (KO) mice have similar blood pressure with varying Na intake but demonstrate attenuated hypertension in response to Ang-II infusion [15]. Further, CD renin KO mice have reduced renal ENaC expression following Ang-II infusion indicating that CD renin modulates BP via regulation of Na reabsorption through ENaC, particularly under conditions with high circulating Ang-II.

While CD renin appears to play a significant role in Ang-II hypertension, its role in Ang-II independent hypertension is currently unknown. We hypothesized that CD renin does not play a role in low Ang-II hypertension. To examine this, we utilized the deoxycorticosterone acetate (DOCA) and high salt model of experimental hypertension. The DOCA-salt model is characterized by low renin. CD specific renin KO mice were not protected from DOCA-salt induced hypertension or renal injury suggesting that CD renin is not likely to be involved in DOCA-salt hypertension.

## Materials and Methods

### Animal Care

All animal studies were conducted with the approval of the Animal Care and Use Committee of the University of Utah in accordance with the National Institutes of Health Guide for the Care and Use of Laboratory Animals.

### Collecting duct renin KO mice

Details on generation of CD specific renin KO mice have been published [15]. In brief, a targeting construct was made containing exon 1 of the *Ren1* gene flanked by two loxP (floxed) sites and electroporated into mouse embryonic stem cells [16]. Mice harboring the floxed exon 1 allele were bred with mice transgenic for aquaporin-2 (AQP2)-Cre to obtain CD specific renin KO mice. All mice were bred on a C57BL/6J background and were bred for over 6 generations. Equal numbers of floxed control littermate and CD renin KO mice of both sexes, aged 3–4 months were used for all studies.

### DOCA-salt protocol and blood pressure monitoring

Floxed control and CD renin KO mice were anesthetized with 2% isoflorane and the right kidney was removed using a dorsal incision. Mice were monitored closely in the post-operative period for signs of pain or distress and treated with Rimadyl wafers as needed. Mice were monitored twice daily for the first 48 hours and then daily thereafter. After 7 days of recovery, radio telemetry devices (TA11-PAC10, Data Sciences International, St. Paul, MN) were inserted with the catheter implanted in the carotid artery. Mice were housed in individual cages, monitored daily for signs of pain or distress and allowed to recover for 5 days before recording BP. No adverse events were noted in the post-operative period following either surgery. After 3 days of baseline BP measurement on normal sodium diet (0.25% Na^+^, Harlan Teklad #2920X, Indianapolis, IN), DOCA pellets (50 mg, 21 day release, Innovative Research of America, Sarasota, FL) were implanted subcutaneously under isoflorane anesthesia. Mice were maintained on a normal sodium pellet diet and provided 0.9% saline for drinking water as per published DOCA-salt protocol [17]. Mice were not handled during BP recording since even small stimuli can markedly affect BP in mice.

### Metabolic balance studies

Following BP measurement for 14 days after DOCA pellet implantation, floxed and CD renin KO mice were placed in metabolic cages for three consecutive days for measurement of food and water intake, body weights and 24 hour urine collection. Mice were fed 9 ml of a gelled diet made from 248 g powdered diet (0.26% Na^+^) (LD101; TestDiet, St. Louis, MO), 14 g gelatin and 110 ml water. Mice were allowed free access to drink 0.9% saline. In a separate experiment, immediately following DOCA pellet implantation, floxed and CD renin KO mice were placed in metabolic cages for 3 days and fed a high salt diet as described above. Note metabolic cage studies were done with the gelled diet instead of the pelleted diet used in the BP studies. Both diets deliver comparable amounts of salt, however the gelled diet is more accurate for assessing total intake in metabolic balance studies, while the pelleted diet is much easier to administer and so is used for the duration of BP studies.

### Plasma and urine analyses

At the end of the metabolic balance studies (day 17 of DOCA-salt), mice were sacrificed and plasma and kidneys were collected for further analyses. Plasma blood urea nitrogen (BUN) was analyzed using a quantitative colorimetric method (BioAssay Systems, Hayward, CA). Plasma renin concentration (PRC) and urinary renin excretion was determined as the amount of angiotensin I (Ang I) synthesized after incubation with excess porcine angiotensinogen using Ang I enzyme immunoassay kit (Peninsula Laboratories, San Carlos, CA). Urinary Na ^+^ and K ^+^ excretion were measured using the Easylyte Analyzer (Medica, Bedford, MA). Urinary albumin excretion was determined with an enzyme linked immunosorbent assay kit (Exocell, Philadelphia, PA).

### Histological analysis for renal fibrosis

Whole kidneys were fixed in 10% formalin, embedded in paraffin and 4 μm sections were obtained. Kidney sections were rehydrated with xylene and ethanol and stained with Masson Trichrome stain or Picro-Sirius solution (Electron Microscopy Sciences, Hatfield, PA). Renal collagen was determined as blue staining (Masson trichrome staining) or red staining (Picro-Sirius staining) under light microscopy. The area of renal fibrosis was calculated by 10 randomly selected non-overlapping fields at 200×magnification using NIH Image J software (National Institutes of Health, Bethesda, MD).

### Quantitative real-time PCR

Renal inner medulla and papilla were dissected from floxed control and CD renin KO mice before and after 17 days of DOCA-salt treatment. Reverse transcription was performed on 1 μg total RNA using the high capacity cDNA reverse transcription kit (Invitrogen, Grand Island, NY). The resulting cDNA was then assayed for relative expression of renin mRNA using a Taqman Gene Expression Assay (renin probe cat. no. Mm02342889_g1, GAPDH probe cat. no. Mm03302249_g1; Applied Biosystems, Carlsbad, CA). Fibrosis in the renal medulla was evaluated using real-time PCR for collagen-1 (probe cat. no. Mm00801666_g1) and neutrophil gelatinase-associated lipocalin (NGAL) (probe cat.no. Mm01324470_m1). Na^+^ transporter mRNA expression in the renal medulla was examined using Taqman primers for epithelial Na^+^ channel (ENaC)-α (probe cat. no. Mm00803386_m1, ENaC-β probe cat. no. Mm00441215_m1, and ENaC-γ probe cat. no. Mm00441228_m1) or SYBR green for Na^+^ chloride cotransporter (NCC Forward primer 5’ TCACCATCAGCACACTGATGGGAGG, reverse primer 5’-ATCACTCCCCAGATGTTGAGCATGCAAC).

### Cell culture

Primary cultures enriched in inner medullary collecting duct (IMCD) cells were prepared from male Sprague-Dawley rats (40–100 g body wt.) as previously described [18]. The IMCD cells grown in 6-well plate were 1% serum-starved for 24 h and then exposed for 24 h to 1 μM aldosterone (Sigma Aldrich, St. Louis, MO) or vehicle or 1 μM Ang-II (Sigma Aldrich, St. Louis, MO). At the end of the experiment, the medium was harvested and applied to MW 10,000 cut-off centrifugal tubes (Amicon Ultra) to concentrate protein higher than ~30 kDa. Renin activity was determined by measurement of ANG I generation in the native condition, active renin content with excess angiotensinogen, and total renin content with excess angiotensinogen and trypsinization as previously described [19]. Prorenin content was the delta value of total renin content and active renin content.

### Statistical analysis

All data are presented as mean ± S.E.M. Student’s t-test was used to compare differences between floxed and CD renin KO mice. ANOVA with repeated measures was used to analyze differences in BP and heart rate between the two groups. Criteria for significance was set as *P*<0.05.

## Results

### Characterization of CD renin KO mice

CD renin KO mice are born at expected frequency, develop normally and have no gross morphological abnormalities. As described previously [15], CD specific deletion of renin was confirmed by genomic PCR for recombination at the renin locus in an organ panel of 10 different organs, quantitative RT-PCR of renin mRNA expression in microdissected CD and measurement of renin activity in the renal medulla and urine.

### DOCA-salt treatment and blood pressure

**Fig 1:**
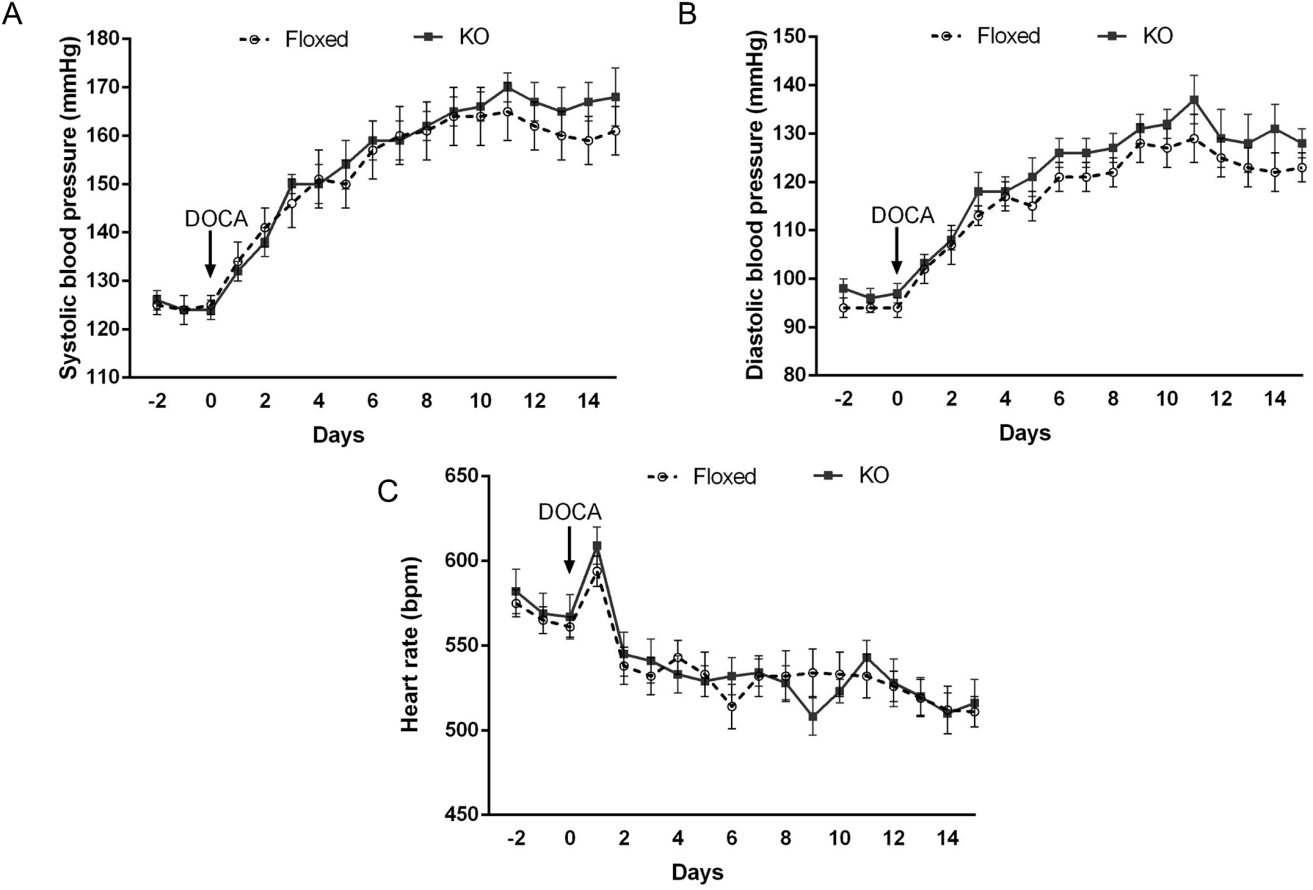
Effect of DOCA-salt treatment on systolic (A) and diastolic (B) blood pressure, and heart rate (C) measured by telemetry in floxed and CD renin KO mice, *N* = 9–10 for each group.

Under baseline conditions, no differences were observed in 24 hour systolic or diastolic BP between floxed and CD renin KO mice fed a normal Na^+^ diet (Fig 1). DOCA-salt treatment increased systolic and diastolic BP to a similar degree in both groups (Fig 1). Similarly, no differences were observed in heart rate between floxed and CD renin KO mice prior to and following DOCA-salt treatment. However, heart rate was lower in both groups at the end of the treatment when compared to baseline (ANOVA P <0.05).

Both floxed and CD renin KO mice had similar food and water consumption, urine volume, body weights after DOCA-salt treatment (Table 1). Urinary electrolyte and water excretion values were analyzed daily during early phase (Days 1–3) and late phase (Days 15–17) of DOCA-salt treatment and were found to be comparable between the two genotypes; values from day 3 and day 17 following DOCA-salt treatment are shown in Table 1.

**Table 1:** Food and water intake, body weight, 24 hour urine volume, urine sodium (UNaV) and potassium excretion (UKV) in DOCA-salt treated floxed and CD renin KO mice. N = 9–10 per group.

	DOCA-salt Day 3		DOCA-salt Day 17	
	Floxed	KO	Floxed	KO
Food intake g/day	3.6 ± 0.2	3.7 ± 0.3	6.0 ± 0.3	6.3 ± 0.4
Water intake, ml/day	12.4 ± 1.5	14.0 ± 0.8	9.0 ± 1.3	9.4 ± 1.3
Weight, g	24.3 ± 1.1	23.4 ± 1.2	28.3 ± 0.6	28.1 ± 0.9
UV, ml/day	8.0 ± 1.3	9.2 ± 0.8	5.6 ± 0.7	5.7 ± 0.7
UNaV, μmol/day	1695 ± 268	2020 ± 248	1122 ± 140	1081 ± 140
UKV, μmol/day	348 ± 13	392 ± 40	356 ± 25	372 ± 22

### DOCA-salt treatment and renal injury

**Fig 2:**
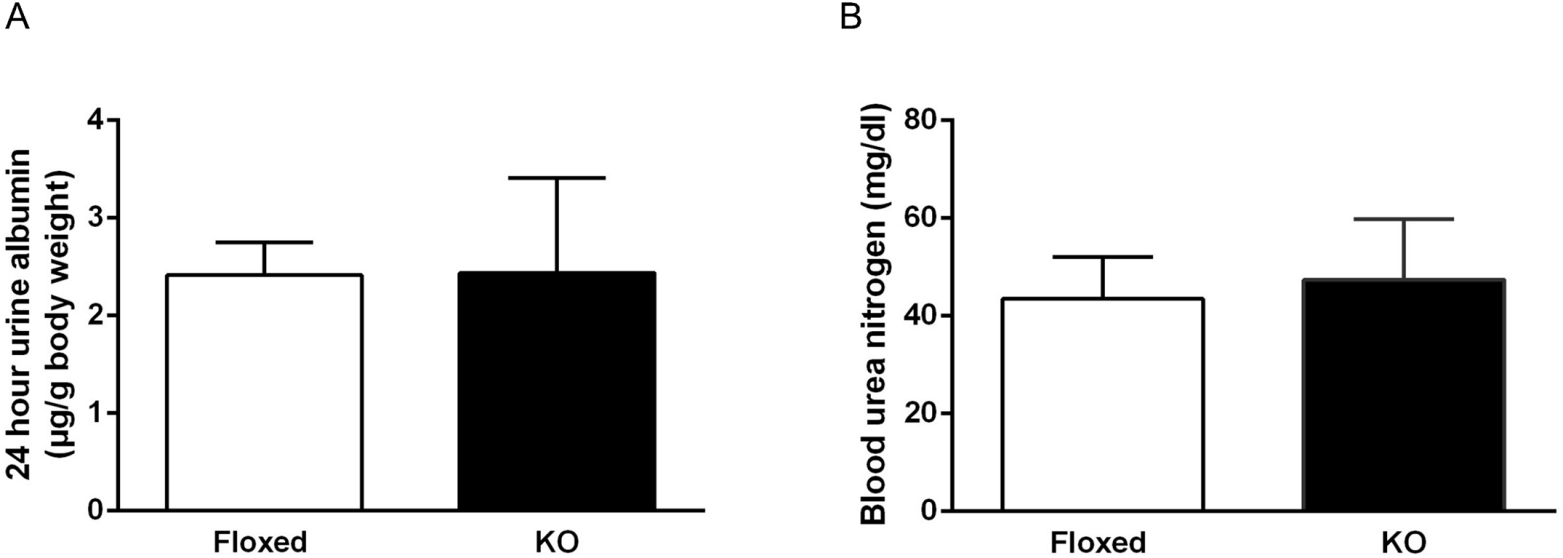
Urine albumin excretion (A) and plasma blood urea nitrogen concentration (BUN) (B) in floxed and CD renin KO mice after 17 days of DOCA-salt treatment, *N* = 9–10 for each group.

**Fig 3:**
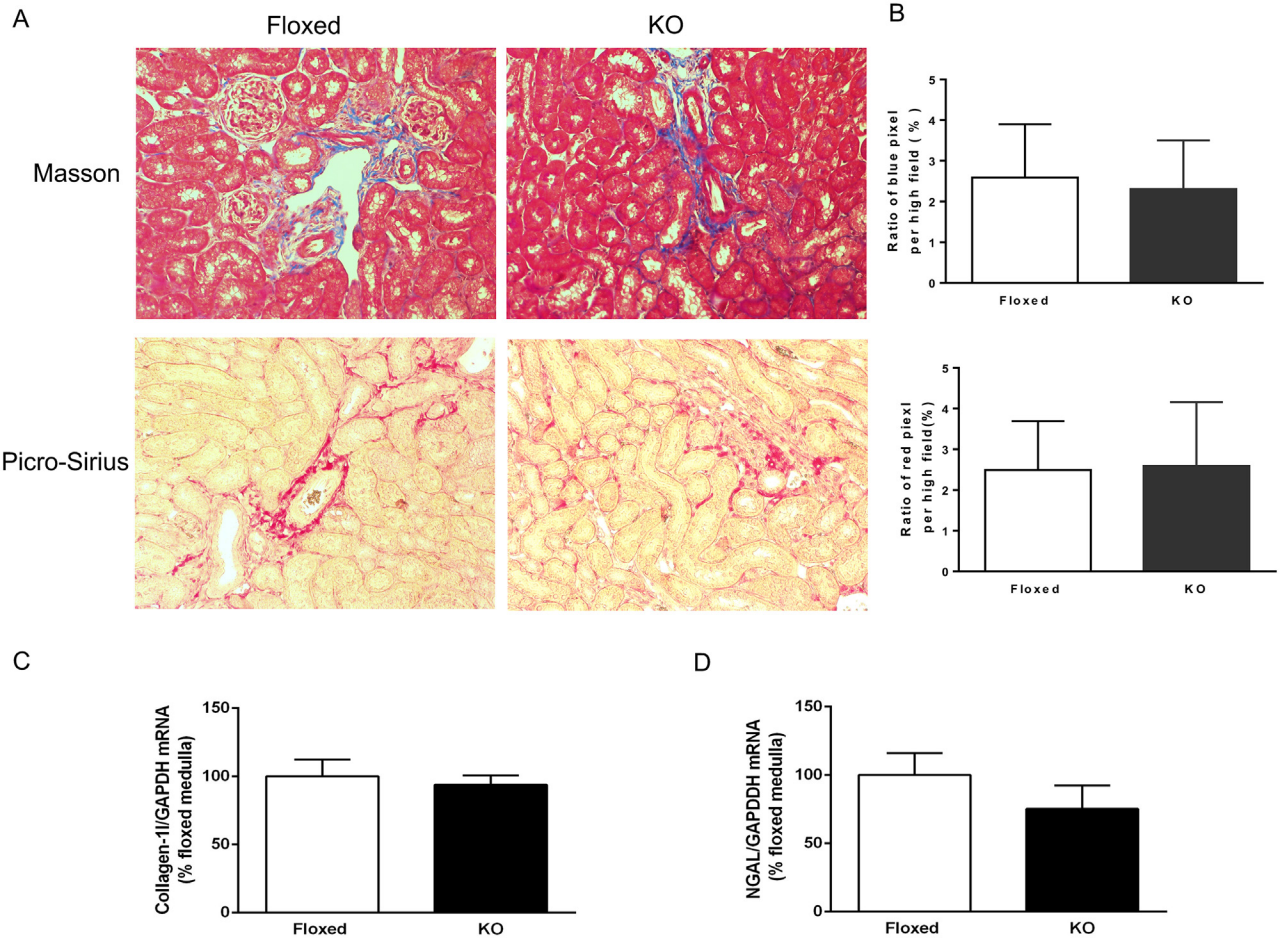
Renal fibrosis in floxed and CD renin KO mice after 17 days of DOCA-salt treatment. (A) Representative images of Masson and Picro-Sirius stain for fibrosis from two different mice. Magnification: × 200. (B) Semi quantitative analysis of the proportion of total area with the blue and red color. (C) Renal medullary collagen-1 mRNA expression. (D) Renal medullary NGAL expression by RT-PCR (N = 4/group).

Compared to floxed mice, CD renin KO mice had similar 24-hour urine albumin excretion and blood urea nitrogen (BUN) levels (Fig 2) following 17 days of DOCA-salt treatment. Renal fibrosis, as assessed by Masson trichrome stain and Picro-Sirius red stain, were comparable between the two groups following 17 days of DOCA-salt treatment (Fig 3A and 3B). Similarly, collagen-1 and NGAL mRNA expression in the renal medulla were comparable between the two groups (Fig 3C and 3D.)

### Effect of DOCA-salt treatment on PRC and urinary renin excretion

**Fig 4:**
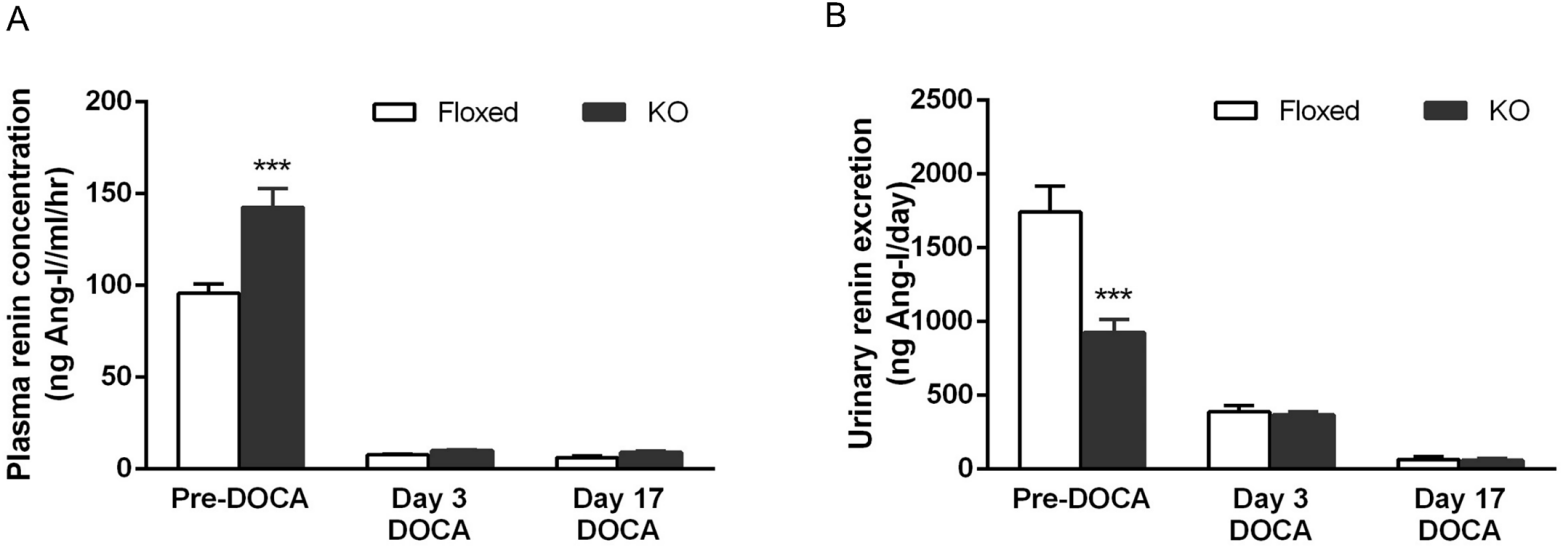
Plasma renin concentration (A) and urine renin excretion (B) in floxed and CD renin KO mice before and after DOCA-salt treatment, *N* = 10–12 for each group. ***P<0.01 compared with floxed mice.

Before DOCA-salt treatment, CD renin KO mice had elevated PRC compared to floxed mice (floxed: 95.7 ± 5.1 vs. KO: 142.6 ± 10.1ng Ang-I/ml/h, *P*<0.01). DOCA-salt treatment suppressed PRC in both groups on days 3 and day 17 following treatment; no differences were observed between the two groups (Fig 4A). Urinary renin excretion was lower in the CD renin KO mice at baseline (925.5 ± 90.5 vs. 1744.6 ± 174.2 ng/day). DOCA-salt treatment reduced urinary renin excretion in both groups on days 3 and 17 without any differences between the two groups (Fig 4B).

### Effect of DOCA-salt treatment on medullary renin mRNA expression

**Fig 5:**
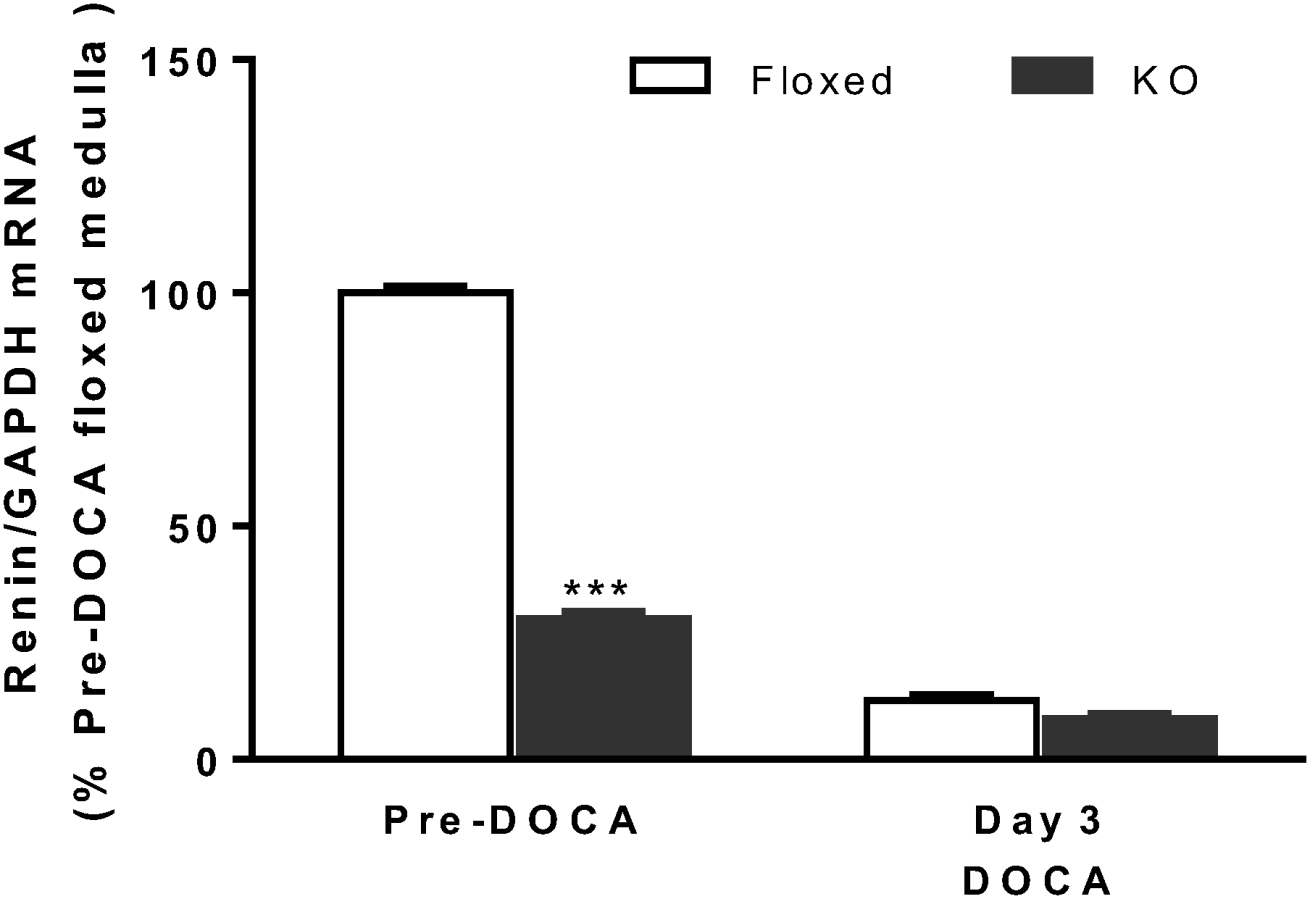
Medullary renin mRNA expression in floxed and CD renin KO mice before and after DOCA-salt treatment, N = 5–9 for each group. ***P<0.01 compared with floxed mice.

In order to determine whether DOCA-salt treatment regulated CD renin synthesis, medullary renin mRNA expression was examined before and following DOCA-salt treatment. At baseline, CD renin KO mice had 70% lower medullary renin mRNA levels compared to floxed mice. DOCA-salt treatment significantly reduced medullary renin mRNA expression in both floxed and KO mice, with no differences detected between the two groups (Fig 5).

### Effect of DOCA-salt treatment on Na^+^ transporter expression

**Fig 6:**
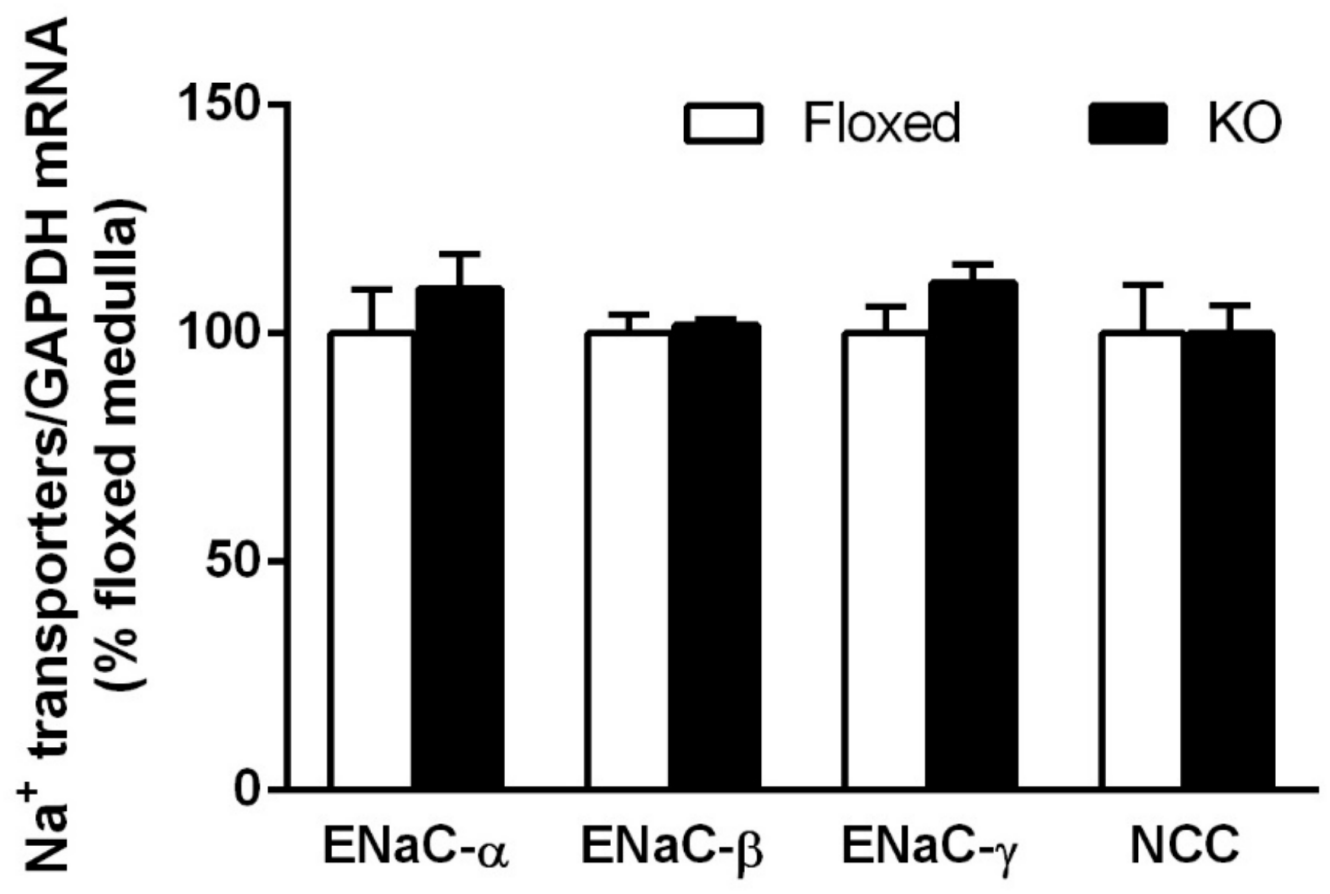
Na^+^ transporter mRNA levels in floxed and CD renin KO mice after 17-days of DOCA-salt treatment. ENaC—epithelial Na^+^ channel, NCC—NaCl cotransporter (N = 4/group).

Both floxed and CD renin KO mice had comparable levels of ENaC and NCC mRNA expression in the renal medulla on day 17 of DOCA-salt treatment (Fig 6).

### Cell culture studies

**Fig 7:**
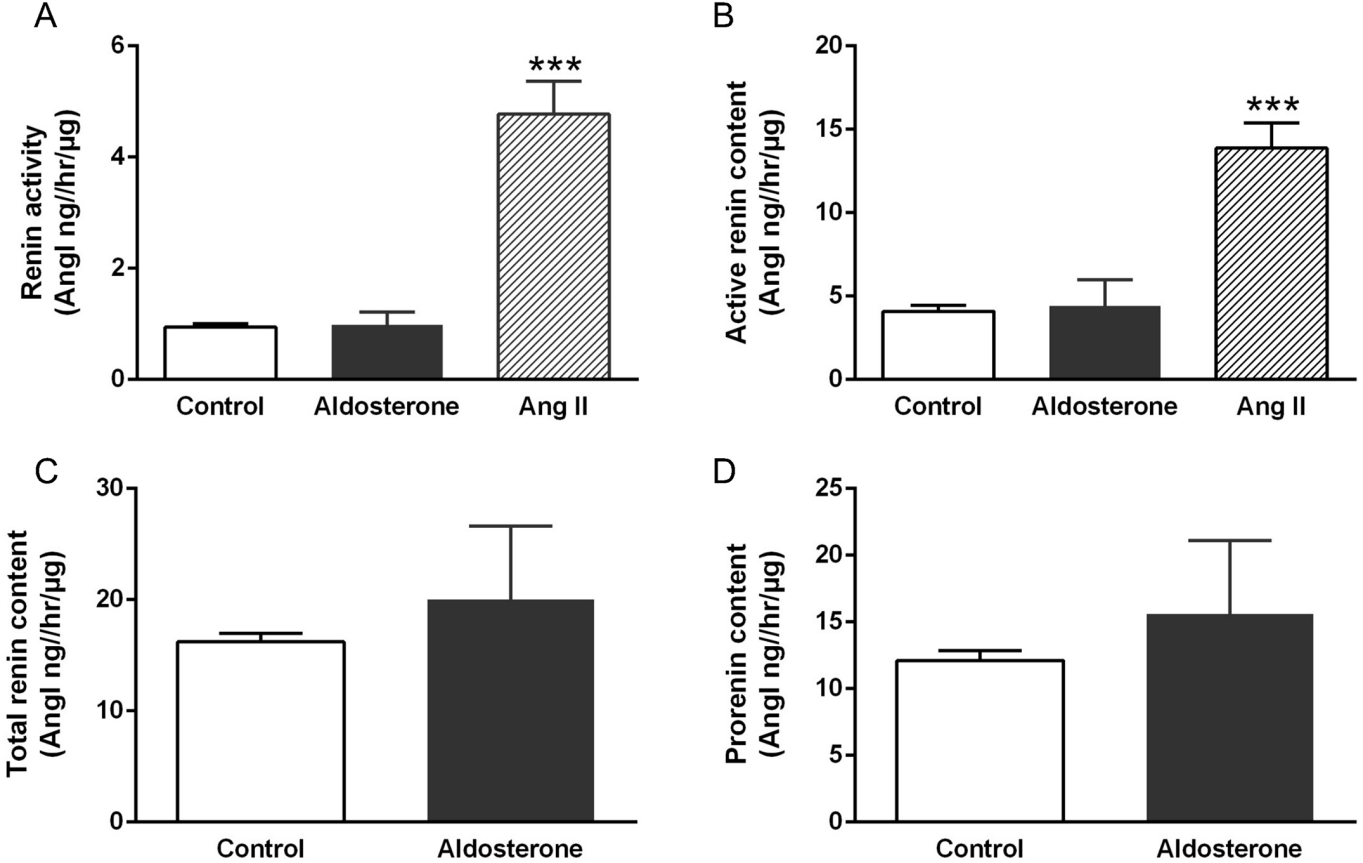
Prorenin/renin activity and content in culture media of primary cultures of rat IMCD treated with 1μM aldosterone, 1 μM Ang-II or vehicle. (A) Renin activity (Ang-I generation in native media), (B) active renin content (measured in the presence of excess angiotensinogen), (C) total renin content (measured in the presence of excess angiotensinogen following trypsinization), and (D) prorenin content (difference between total and active renin content). N = 8–10 for each group. ***P<0.01 compared to vehicle.

Treatment with aldosterone for 24 hours did not change renin activity in media of primary cultures from rat inner medullary CD. Similarly, no differences were observed in active renin (measured in the presence of excess angiotensinogen) or total renin content (measured in the presence of excess angiotensinogen following trypsinization) between the control and aldosterone treated groups (Fig 7). In contrast, treatment with Ang-II increased renin activity and active renin content in primary culture media.

## Discussion

The main objective of the current study was to examine whether CD renin KO attenuates hypertension and renal injury in DOCA-salt hypertension. Compared to floxed mice, CD renin KO mice had no differences in BP or heart rate with DOCA-salt treatment. These findings are in contrast to that of Ang-II infused hypertension, wherein selective ablation of CD renin significantly attenuated the hypertensive response [15]. The reasons for the varied role of CD renin in Ang-II and DOCA-salt hypertension are not fully known, however differing effects of these agents on the intrarenal RAS may be involved. Our study found that CD derived renin, like JGA renin is suppressed with DOCA-salt treatment. These results are consistent with a previous study which demonstrated that renal mRNA expression of angiotensinogen, renin and angiotensin converting enzyme were reduced in DOCA-salt treated rats [20]. Further, systemic administration of RAS blockers does not prevent hypertension in rodents treated with DOCA-salt [21–23]. In contrast, Ang-II infusion increases renal production of angiotensinogen and CD production of renin [9, 24] and is prevented by renal-selective RAS knockdown [15, 25–27]. Taken together, the above supports the notion that the hypertensive effects of Ang-II, but not DOCA-salt, involve the intra-renal RAS.

Despite the lack of an antihypertensive effect, RAS blockers have been shown to ameliorate renal damage and cardiac hypertrophy following DOCA-salt treatment [21–23]. However, proteinuria and renal fibrosis were identical in floxed and CD renin KO mice in the current study. One explanation for this discrepancy might be related to RAS blockers improving renal blood flow and vascular resistance by modulating efferent arteriolar vasodilation [28, 29], which may not be altered by CD renin. It is also possible that RAS blockers might work through mechanisms independent of RAS blockade, e.g. activation of angiotensin type 2 receptors (mice with global deletion of angiotensin type 2 receptor demonstrated a steeper and greater rise in BP with DOCA-salt treatment compared to controls) [30]. Regardless of the mechanisms by which RAS blockade attenuates renal injury, it is clear that CD renin is not involved in mediating renal injury in DOCA-salt hypertension.

Unlike Ang-II, which augments renal medullary renin synthesis, DOCA-salt suppressed renal medullary renin. Such a response is physiologically appropriate and needed to counterbalance the sodium retention and volume expansion mediated by DOCA-salt treatment. Compared to baseline, both floxed and CD renin KO mice demonstrated enhanced natriuresis as early as day 3 following DOCA-salt treatment. However, as with BP or renal injury, no differences were observed between the two groups. Further, ENaC and NCC mRNA levels were comparable between the two groups. In accordance with the *in-vivo* data, treatment with aldosterone did not alter total or active renin content in media of primary cultures of rat IMCD. Collectively, these results suggest that aldosterone does not stimulate CD renin synthesis. Whether other components of the intra-renal RAS (e.g., the angiotensin receptor type 2) are activated by aldosterone is currently unknown.

Of note, PRC in this study was at the lower end of normal PRC values reported in mice (0.1–3.4 μg Ang-I/ml/hr) [31], even before DOCA-salt treatment. One potential explanation for this finding might be related to the substrate (porcine versus rat angiotensinogen) used to measure renin concentration; mouse renin reacts with rat angiotensinogen 10 times faster with greater Ang-I generation than mouse or porcine angiotensinogen [32]. Therefore, the values in this study are likely to be lower than if rat angiotensinogen was used to measure renin concentration. Nevertheless, no differences were observed between the two groups following DOCA-salt treatment.

The current study has potential limitations. Prior to DOCA-salt administration, medullary renin mRNA levels were reduced by about 70% in the CD renin KO mice suggesting that not all renin-producing cells might be targeted in our model. However, as shown in our previous study, renin mRNA levels in micro-dissected cortical CD, papilla and inner medulla were markedly reduced (>90%) in the CD renin KO mice compared to controls [15]. Secondly, when exon 1 of the renin gene (which contains the transcriptional start site) is deleted, an alternative transcription site in intron 1 might lead to renin synthesis. However, this renin lacks the signaling peptide, remains intracellular and is predominantly found in the brain [16]. The current study did not evaluate if intra-cellular renin might contribute to DOCA-salt hypertension.

A key remaining question pertains to the role of CD renin in other forms of Ang-II independent hypertension. Some studies propose that the intra-renal RAS may be involved in hypertension induced by inhibition of nitric oxide (NO) synthesis with *Nω*-nitro-l-arginine methyl ester (L-NAME) [33, 34]. L-NAME induced hypertension is characterized by low circulating plasma renin and Ang-II levels [35] and is attenuated by treatment with RAS blockers [36, 37]. Further, renal expression of angiotensinogen, angiotensin converting enzyme and angiotensin type 1 receptor were increased in L-NAME treated rats compared to controls [33]. Consequently, CD renin might also be up regulated in L-NAME hypertension. Additionally, a recent study reported that absence of angiotensin converting enzyme selectively within the kidney protects against L-NAME induced hypertension [34]. Future studies utilizing the L-NAME model and other experimental hypertension models are needed to clarify the functional significance of CD renin in Ang-II independent hypertension.

In summary, the current study demonstrates that CD renin is not essential to the hypertension or renal injury associated with DOCA-salt treatment, which is one form of low Ang-II hypertension. Whether CD renin is important in other forms of Ang-II independent hypertension remains to be determined.

## Supporting Information

S1 Data Additional information for each figure of the manuscript is available in the supporting dataset. (XLSX)
